# Transforming air pollution and health research into action in low- and middle-income countries

**DOI:** 10.1097/EE9.0000000000000434

**Published:** 2025-11-07

**Authors:** Jonathan Samet, Kerolyn Shairsingh, Wenlu Ye, Sophie Gumy, Pierpaolo Mudu, Zorana Andersen, Wei Huang, Michal Krzyzanowski, Sumi Mehta, Helen Petach, Annette Peters, Ajay Pillarisetti, Jason West, Caradee Y Wright, Thomas Clasen

**Affiliations:** a Colorado School of Public Health, Aurora, Colorado; b World Health Organization, Geneva, Switzerland; c University of Copenhagen, Copenhagen, Denmark; d Peking University, Beijing, China; e Imperial College London, London, United Kingdom; f Vital Strategies, New York City, New York; g United States Agency for International Development, Washington, DC; h Helmholtz Munich, Oberschleißheim, Germany; i University of California Berkeley, Berkeley, California; j University of North Carolina, Chapel Hill, Chapel Hill, North Carolina; k South African Medical Research Council, Cape Town, South Africa; l University of Pretoria, Pretoria, South Africa; m Emory University, Atlanta, Georgia

**Keywords:** Air pollution, Low- and middle-income countries, Health research, Frameworks

## Abstract

This commentary highlights the need for actionable and context-appropriate research on air pollution and health that will continue to drive policies to reduce exposures and disease burden. Research on air pollution and health has been substantial in high-income countries (HIC), leading to causal conclusions on the adverse effects of air pollution. Despite bearing the greatest disease burden from air pollution, low- and middle-income countries (LMICs) have had scant research funding, a trend that may well be aggravated due to changing political priorities in some HICs. High-quality data from LMICs is urgently needed to help motivate local, subnational, and national policies to raise awareness and identify priority actions to improve health. The new evidence will also provide a more complete understanding of air pollution and health globally. We highlight a framework for moving from research to action and address how this framework differs in HIC and LMIC contexts. We propose a hierarchy of research needs that begins with having the necessary air pollution monitoring and health data, and the capacity to use the data for informative analytics, risk assessment, valuation, and policy formulation. Building technical capacity may be needed for this purpose, as will development of a functioning regulatory system in parallel. We call for greater emphasis on surveillance studies to demonstrate the benefits of action and address barriers to action. The global community would benefit from a broad research agenda with priorities and adequate funding dedicated to building evidence that leads to positive policy change. We urge priority for advancing actionable research and improving research capacity in LMICs, including investments in routine collection of relevant data, emphasizing the foundation of risk monitoring and health data systems, and building a cadre of researchers and informed policy-makers.

**What this study adds**

This commentary highlights the need for actionable and context-appropriate research on air pollution and health that will continue to drive policies to reduce exposure and disease burden. We highlight a framework for moving from research to action, and address how this framework differs in high-income countries and low- and middle-income countries contexts. We also propose a hierarchy of research needs that begins with having the necessary air pollution monitoring and health data, and the capacity to utilize the data for informative analytics, risk assessment, valuation, and policy formulation.

## Introduction

**Figure 1.**
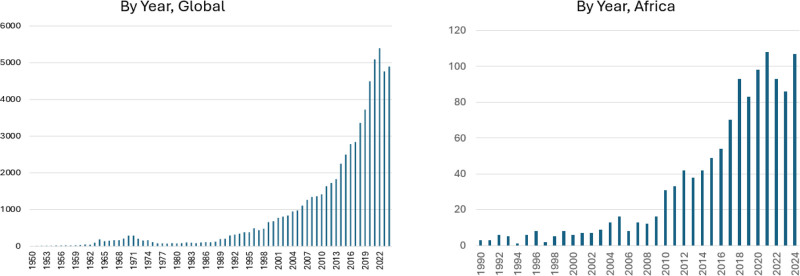
Annual number of global (1950–2024) and African (1990–2024) publications found with PubMed search on “air pollution and health.”

While research on air pollution and health has been substantial in high-income countries (HICs), it lags in low- and middle-income countries (LMICs).^1^ From 1950 through 2024, an estimated 70,000 studies were published on air pollution and health. The pace of publication has accelerated, reaching 5,000 per year. These studies include only modest numbers from LMICs in Eastern Europe, Latin America, Sub-Saharan Africa, and South Asia, where the burden of disease from air pollution is among the highest. However, an encouraging trend of growth, with 200 publications in 2024, can be seen in Africa (Figure 1). Still, the cumulative number of papers from Africa is only 1,200. Despite the myriad research studies conducted since the London Fog of 1952 and other disasters that rang the air pollution alarm bell, only about half (97) of the world’s countries, primarily HICs, have national air quality standards for particulate matter, a critical policy for a country to protect its citizens from the risks of ambient air pollution.^2,3^

The available research findings should be sufficient to motivate implementation of effective standards, particularly as particulate matter air pollution is the leading environmental contributor to disease burden, with millions of attributable deaths.^4^ However, contributing to the regulatory void in LMICs is a lack of locally relevant research evidence needed to motivate and set national standards. While global evidence of the health impacts of air pollution is synthesized by the World Health Organization (WHO) in its Global Air Quality Guidelines (AQGs), nationally derived research evidence can strengthen the motivation for setting national standards.^5^

Gaps in research evidence have been identified on air pollution and health by various committees and researchers. There are clear targets for investigation: enhancing monitoring methods, reducing uncertainties in exposure estimation, characterizing health risks, assessing synergies of pollutants with climate change, and evaluating impacts of regulatory standards and policies. Relevant to air pollution control, studies have highlighted gaps in understanding of the health impacts from source sector-specific pollution across different microenvironments. For particulate matter specifically, investigations addressing sources of particulate matter require data on chemical composition and particle number/size distribution, and population activities, along with improved emission inventories and high-resolution models^6–8^

This commentary emphasizes the need to move from research to action. We propose a hierarchy of research needs to generate evidence that will motivate and guide action. The hierarchy reflects the differing contexts of countries, spanning from LMICs to HICs.

### The connection between research evidence and policy action

Research is an essential ingredient to setting regulations for air quality management. It is needed to identify what environmental compounds are causing harm to human health and what levels of risk they convey. Research evidence guides the development of risk assessment and implementation of policies to reduce exposure to pollutants. So-called accountability research is then needed to assess whether there are changes in exposures and benefits in terms of promised reductions in disease burdens from policy measures.

Research findings have been the basis for promulgating evidence-based air quality guidelines and standards at global levels. The WHO AQGs systematically draw on the scientific literature, leveraging global experts to conduct systematic reviews and quantitative analyses of health studies. The AQGs, which are revised regularly based on new research, help inform national standards, such as those of the European Union.^9^ As the intent in formulating air quality standards is to set them at achievable levels that provide an acceptable level of risk to health and the environment while encouraging incremental national improvements, continuous surveillance can inform when the standards have been achieved and when improvements can be made.

We highlight a strategic framework that connects the identification of research needs with the development of evidence-based actions. The process moves from research to regulation and is ongoing and iterative, with surveillance studies supporting a feedback loop. While idealized, the framework is a conceptual vehicle for considering how research informs policy development and implementation for air pollution regulations. It offers a tool for judging how the findings of a study will fit into the policy process: providing evidence to inform the selection of the level of a standard or evaluating the consequences of a standard.

**Figure 2.**
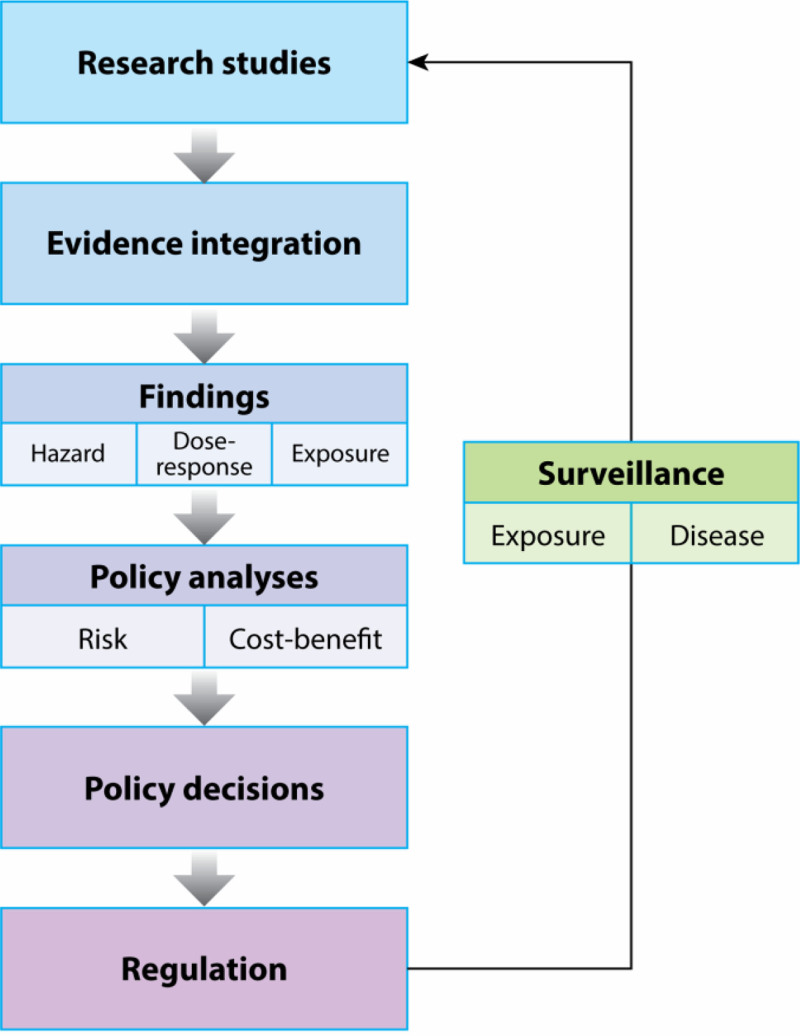
General framework for moving from research to policy action.^9^

**Figure 2** highlights these steps: (1) research studies, (2) evidence integration, (3) findings, (4) policy analysis, (5) policy decisions, (6) regulations, and (7) surveillance.^9^ The first step, carrying out research, can be made strategic by targeting local areas of uncertainty in exposure and health assessments. The synthesis and integration of this research evidence for decision-making can be a resource-intensive step and generally involves expert judgment and institutional support. Policy analyses and decisions draw on the evidence integration and findings and may incorporate risk and cost assessments to determine the optimal policy action. Surveillance studies assess the policy impact (e.g., change in air pollution levels or reduction of adverse health outcomes) and provide guidance on what additional research is needed to make the policy action more effective.

## A hierarchy of research needs

**Figure 3.**
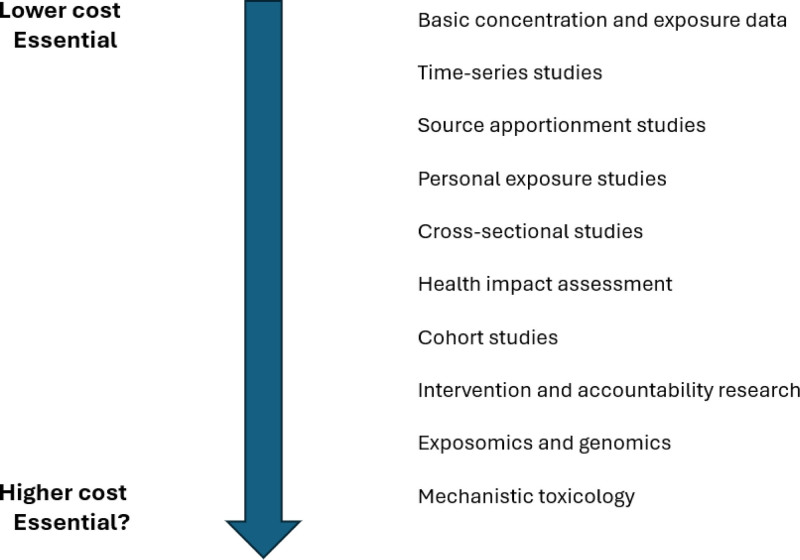
Hierarchy of research needs.

We propose that research be targeted along a hierarchy of needs and research questions that highlight important differences between LMICs and HICs based on resources and immediate priorities (Figure 3). Of necessity, the diagram oversimplifies, and the range of costs from “lower” to “higher” is qualitative. In HICs, increasing public alarm over the health effects of air pollution long ago prompted calls for research that could inform effective control strategies. Air quality measurements and health risk estimates are the core inputs for quantifying the disease burden. With the benefit of extensive resources, regulatory air quality measurements are sufficiently dense to provide exposure estimates for health impact assessments. Extensive research (Figure 1) has linked air pollution to numerous adverse health outcomes and shown risk at prevalent levels of particulate matter in HICs. This robust literature, primarily from studies in North America, Europe, and China, underlies burden of disease estimates. The conduct of these studies is facilitated by the availability of large health databases, some at the national level.

While these studies in HICs and some LMICs are expanding the list of adverse outcomes linked to air pollution, more research is needed into determinants of toxicity of air pollution mixtures, mechanistic models, and critical uncertainties in exposure assessment. For example, research that provides deeper insights into sources and particle-and-gas phase characteristics of particulate matter (PM) can guide the development of refined standards and policies that have the greatest health impacts. Long-standing questions about the toxicity-determining characteristics of PM have not yet been answered with certainty. While evidence on the health burden from source-specific contributions, such as coal-fired power plants, wildfires, and sand and dust storms, is emerging, more knowledge is needed.^10–12^ Ongoing research is also needed to address the multipollutant nature of air pollution that can vary drastically across different microenvironments. Epidemiological research continues to grapple with the handling of the multipollutant nature of air pollution and characterizing mixture exposures to minimize misclassification with the goal of better characterizing the population exposure and associated health impacts.^13,14^

The research priorities in LMICs are generally quite different. Rapid industrialization, urbanization, transportation growth, agricultural practices, and, in some instances, loose regulation are contributing to increases in air pollution.^15^ Long-standing sources, such as biomass smoke and open refuse burning, contribute to ambient air pollution, while cooking and heating with wood, charcoal, and dung, and lighting with kerosene cause a substantial disease burden from household air pollution. These contrasts with HICs lead to uncertainty in extending the findings from HICs to LMIC settings and undermine the local credibility of burden estimates. Unfortunately, many LMICs lack the basic substrate for air pollution research, including emission and exposure monitoring, health databases on basic outcomes, and trained and funded researchers. For example, air pollution monitoring for PM_2.5_ is sparse or lacking in many countries of Sub-Saharan Africa, and mortality and morbidity health data are not collected nor available through accessible databases.^16^ In the WHO African region, with its high disease burden from air pollution, 91% of the death registration data is of low quality because the needed systems are not in place. To inform local policy actions, accessible local health data are needed, but obtaining data is challenging, absent electronic systems. For example, investigators in the Eastern Africa GEOHealth Hub (Ethiopia, Uganda, and Kenya) are manually extracting data from hospital admission records to carry out time-series studies of PM_2.5_ and hospitalization.^17^

Accurate monitoring of pollutant concentrations is fundamental; where possible, monitoring of PM_2.5_ with an internationally recognized reference-grade monitor is an important starting point.^18,19^ While multiple monitors across a location are preferable, a single monitor sited correctly will both provide information on the severity of air pollution and support certain types of research, as well as calibration of low-cost sensors. Mobile monitoring platforms (e.g., vehicles with instruments) have also been used by countries to complement regulatory monitors and enhance mapping of the spatial distribution of pollutants, and to carry out specific ad hoc sampling campaigns.^20^ As routinely collected reference-grade data are limited in many LMICs, ground-level measurements are often supplemented with additional data sources, such as low-cost sensors, satellite observations, and emissions models.^21,22^ As estimates from satellite data become more refined and better understood in LMIC settings, they should become increasingly useful but will need validation against ground measurements to be credible. Emission estimates can be made for important point sources using proxies such as oil and gas consumption, motor vehicle usage, and industrial production.^23^

In the LMIC setting, the joint effects of ambient and household air pollution need to be addressed. Mortality from the joint effects of ambient and household air pollution is highest in LMICs, especially in South Asia, sub-Saharan Africa, North Africa, and the Middle East, where limited exposure and health risk data are available. Thus, research on ambient air pollution needs to be coupled with research on household air pollution, recognizing that for a substantial fraction of the population in some countries, exposure to air pollution occurs from both ambient and household exposures. Research on household air pollution has explored a broad range of questions: (1) What are the exposure levels associated with household polluting fuels burnt in various devices across different settings? (2) Are adverse health effects associated with household air pollution exposure from burning polluting fuels with inefficient technologies? (3) What can be done to reduce emissions and exposures? (4) Will alternatives to the combustion of polluting fuels be accepted and utilized? and (5) Do alternative fuels and cooking/heating technologies reduce health risks? Research needs on household air pollution vary with country context and relate to generating evidence to support substitution of alternative stoves and fuels, establishing the feasibility and acceptability of selected approaches, figuring out how best to scale affordable, acceptable, and effective clean household energy, and tracking the short- and long-term impacts of interventions.

In all settings, research should also consider the intersection of health, air pollution, and climate change. Warmer temperatures and drought will increase the frequency and severity of wildfires and sand dust storms, a consequence already being experienced.^24^ There will also be episodes of extreme heat that coincide with high air pollution as warmer temperatures increase ozone concentrations. While health research has focused on air pollution and climate change individually, more research is needed on the impact of joint exposures, for example, to heat and air pollution. More knowledge is also needed on the long-term effects of exposure to particles from wildfires and sand and dust storms, as these extreme events increase with climate change.^25^

### Implementing policy action and surveillance

Within the framework of Figure 2, policy research and analyses are also needed to examine governance around air pollution control and barriers to moving forward with the implementation of guidelines and standards. Critical questions that should be explored include: (1) What are the barriers to moving forward in the process? (2) What stakeholders can act as drivers and push the process forward? and (3) Are there critical gaps for which research could make a difference? Relevant approaches include assessment of the structures and policies in place, stakeholder mapping, and evaluation of the public’s knowledge and attitudes concerning air pollution. Such research is distinct from what most public health researchers do, and it would be facilitated by partnering with policy researchers and decision-makers.

Air pollution control comes with the implicit promise that public health will benefit through the reduction of air pollution exposure. To objectively evaluate the success and limitations of the policy action after implementation, the observed changes in exposure and health should be compared to projected changes.^26^ The implementation of a policy does not always result in the outcomes intended. Implementation research can clarify why interventions may fail to transfer from one country to another. This research can focus on implementation processes and results as well as local factors (e.g., such as, social, cultural, economic, and political considerations) that can lead to contextual differences in outcomes. To assess how well implementation has occurred, researchers can explore questions on the acceptability, adoption, appropriateness, feasibility, implementation cost, and sustainability of the implementation strategy. Because air pollution affects everyone, it is beneficial to conduct participatory action research, which gives participants power and control over the process and allows them to be involved in the identification, design, and implementation phases of research and not just be targets for the dissemination of study results.

Policy surveillance studies align well with the assessment of effectiveness, and both require advance planning to ensure that the needed data will be available.^27^ In data-rich countries, these types of studies can be conducted even if policies were not planned with the intention of long-term impact analysis. The health benefits of national programs, such as policies directed at industrial or transportation emissions or agricultural burning, can be evaluated in surveillance studies using emergency room visits. Interventions to promote cleaner cooking fuels such as liquified petroleum gas and electricity can be assessed using experimental study designs such as randomized and quasi-randomized controlled trials. These differences in sources of pollution and data collection require tailored study designs and analytical approaches for LMICs. Recognizing that air pollution is influenced by myriad actors, it is critical to collect data on air pollution and health outcomes before and after the intervention for an adequate time-period, when possible, to disentangle the true effectiveness of the intervention from other changes, for example, from changes in meteorology.

To maximize the co-benefits of policy action, both air pollution and climate change impacts should be evaluated. While some air pollution policies may be beneficial in mitigating climate change, others may not. For policy actions that lead to improved climate outcomes but increase risks to health, or vice versa, additional analyses are needed to weigh costs and benefits and assess where the balance lies. It is important to highlight that the output from policy surveillance studies should not only be viewed as offering lessons learnt but also future research recommendations to build the evidence base for better policy action.

### Developing capacity and funding for research

Needs for research and technical capacity-building vary widely, with some countries having established research centers and others lacking trained researchers. We suggest an inventory of training needs, perhaps carried out by the WHO, and an organized approach to filling gaps in capacity to conduct exposure and health impact assessments. The Health Effects Institute’s Public Health and Air Pollution in Asia Project was a collaborative set of multi-city time-series studies that used a shared protocol to standardize exposure and health data collection. The studies provided regionally relevant results while serving as a capacity-building opportunity.^28^ This model can be emulated and fostered by agencies and governments supporting air pollution research in LMICs. In situations where resources are limited, piecemeal funding could support participation in international collaborations, such as the Multi-Country Multi-City research initiative, which has proved successful even without significant funding, reflecting dedicated leadership by the coordinating investigators and the enthusiasm of researchers around the globe. These collaborative research networks are also excellent for providing training to the next generation of researchers.^29^

Stating the obvious, carrying out research requires funding. Air pollution research is chronically underfunded, given the disease burden and the investments in fossil fuels compared with clean air initiatives. In HICs, support for air pollution and climate research comes primarily from science and environment agencies. While the United States has traditionally been a major funder of environmental health research, rollbacks in governmental funding will significantly reduce financial support both nationally and abroad, while also making it difficult to access key environmental databases. China and the European Union are also major funders of air pollution research, though it is unclear whether they will increase funding to address emerging research needs. Foundations such as the Wellcome Trust support research on climate change and health, while the Health Effects Institute funds epidemiological studies, and regional capacity building and accountability studies.^30^ Bloomberg Philanthropies also supports research on air pollution mitigation, especially in cities. The private sector may also be a source of funding, provided researchers can remain independent and shielded from commercial interests.

Funding priorities in LMICs have emphasized the building of research capacity. One example is the Science and Technology Division of the African Union funding programmes, which aim to (1) accelerate Africa’s knowledge-based economy and research capacity, (2) promote greater involvement of African scientists, researchers, and institutions in implementing action plans, and (3) improve international collaboration. Using its convening power, the WHO can bring political leadership and scientific expertise together to create an agenda that might prioritize research that will yield actionable evidence and address accountability.

The same political forces that are constraining resources for air pollution research make it even more important to pursue such research using creative approaches. Even a temporary pause in research will force some investigators to pivot to other work and create opportunities for industry to fill the gap. Where possible, investigators will need to employ study designs that minimize field and lab costs, expand the use of existing data, develop efficient and informative statistical methods, and take great advantage of machine learning and artificial intelligence. Senior researchers could redouble efforts to support early-career and LMIC investigators. Leveraging institutional cooperation and ongoing data collections for other goals can maximize research outputs. The relevance of research could also be enhanced by collaborations of researchers with decision-makers to ensure that research is policy-relevant and actionable.

## Conclusions

The 2025 WHO Air Pollution Conference offered a venue for discussion of next steps in research and action on air pollution globally. It brought together policy-makers, research funders, researchers, and other stakeholders, such as medical associations, patient organizations, and environmental Non-Governmental Organizations. Progress on improving air quality has been made in HICs, and that progress could be accelerated by steps taken for climate change mitigation. Over the last decade, there has been increased emphasis on building technical and research capacity in LMICs and, partially as an outcome of these efforts, the body of relevant exposure and epidemiological evidence is growing. Dissemination of research findings to mainstream media is also critical to raise awareness among the public and put pressure on politicians by demanding clean air actions and influencing the flow of funds. We urge priority for advancing research and technical capacity in this context, emphasizing the foundation of monitoring air quality and health data systems and building a cadre of researchers, informed and empowered citizens, and policy-makers who will work together towards cleaner air for all.

## Conflicts of interest statement

The authors declare that they have no conflicts of interest with regard to the context of this report.
